# Anticoagulant Effect of Snow mountain garlic: *In Vitro* Evaluation of Aqueous Extract

**DOI:** 10.3390/molecules29204958

**Published:** 2024-10-20

**Authors:** Isabel Clark-Montoya, Yolanda Terán-Figueroa, Denisse de Loera, Darío Gaytán-Hernández, Jorge Alejandro Alegría-Torres, Rosa del Carmen Milán-Segovia

**Affiliations:** 1 Faculty of Chemical Sciences, Autonomous University of San Luis Potosi, Dr. Manuel Nava Martínez Avenue #6, University Zone, San Luis Potosi 78210, Mexico; a201618@alumnos.uaslp.mx (I.C.-M.); atenea.deloera@uaslp.mx (D.d.L.); 2 Faculty of Nursing and Nutrition, Autonomous University of San Luis Potosi, Niño Artillero Avenue #130, University Zone, San Luis Potosi 78240, Mexico; yolandat@uaslp.mx (Y.T.-F.); dgaytan@uaslp.mx (D.G.-H.); 3 Department of Pharmacy, University of Guanajuato, Noria Alta s/n, Building I, Guanajuato 36050, Mexico; ja.alegriatorres@ugto.mx

**Keywords:** thromboembolic disease, *Allium ampeloprasum* L., Snow mountain garlic, *in vitro* effect, human plasma, isoflavones

## Abstract

Snow mountain garlic is traditionally eaten by Himalayan locals for its medicinal properties. Although different species of the genus *Allium* are known to have other biological effects, such as antiplatelet and antithrombotic activities, little is known about the anticoagulant effect of Snow mountain garlic, a member of the genus *Allium*. Therefore, the present study examined the *in vitro* anticoagulant effect of the aqueous extract, the lyophilized aqueous extract, and the isoflavone extract from the lyophilized aqueous extract of Snow mountain garlic in samples from 50 human blood donors. Compared to the control, concentrations of 25, 12.5, and 6.25 mg/100 µL lengthened the clotting times of prothrombin, and concentrations of 25 and 12.5 mg/100 µL lengthened the activated partial thromboplastin time (*p* ˂ 0.05). The isoflavone extract from the lyophilized aqueous extract containing isoflavones, organosulfur compounds, a polyphenol, and a steroid glycoside showed a significant effect (*p* ˂ 0.05) on the prothrombin time and the activated partial thromboplastin time at a dose of 20 µL (volume) compared to the control. The results regarding the use of Snow mountain garlic as a preventive measure and aid in treating thromboembolic disease are promising.

## 1. Introduction

Thrombosis is the third highest cause of death worldwide, characterized by vascular occlusion, as in ischemic heart disease (myocardial infarction), venous thromboembolism, and cerebrovascular disease [1]. In Mexico, 1052 cases of ischemic heart disease were reported (653 in men and 399 in women), along with 994 cases of cerebrovascular disease (557 in men and 437 in women) and 2051 cases of peripheral venous insufficiency (661 in men and 1490 in women). In January 2023, 995, 1097, and 2666 cases were reported, respectively [2]. These data indicate that we are facing a public health problem that requires close attention, as patients’ lives are at risk.

The anticoagulants available to the public are currently effective in preventing and treating disorders such as ischemia, cerebrovascular accidents, deep vein thrombosis, and pulmonary embolism. However, their use has drawbacks, such as the need for monitoring. Some must be administered intravenously, making their long-term use impractical. Others are administered orally but have a variable anticoagulant response, due to various factors ranging from the patient’s genetic makeup to the intake of foods containing vitamin K. This variability requires frequent dose adjustments to ensure the therapeutic effect and avoid bleeding [3,4,5]. Additionally, side or undesirable effects may be caused by commercially available medications, such as gastric bleeding, duodenal ulcers, and hemorrhages [6]. The key is to obtain or develop new anticoagulant therapies. There is ongoing research to find new treatments for managing thromboembolic diseases that are safer [7], accessible, and affordable.

The use of herbs as remedies to treat diseases is as old as humanity itself. It has been reported that plants played a significant role in curing illnesses in ancient cultures such as the Egyptian, Greek, and Chinese civilizations. It is important to remember that several medicines currently used to treat human diseases have been derived from plants [8]. With the passage of time, industrialization, and scientific and technological progress, the knowledge of medicinal plants has remained within specific groups of people, passed down from generation to generation among the traditional doctors of indigenous peoples [9].

At present, interest in and attention to extracts obtained from various plants used in traditional medicine have increased, focusing mainly on the pharmacological activity of their molecules or phytoconstituents such as sterols, terpenoids, alkaloids, flavonoids, saponins, and tannins [10,11]. These compounds have demonstrated anti-inflammatory, analgesic, antidiabetic, or anticancer properties, among others [11,12,13].

An example of this is Snow mountain garlic, traditionally consumed by locals in the Himalayas for its medicinal properties. They use it to increase energy levels, detoxify their bodies, and mitigate rheumatoid arthritis. This important herb grows at 6000 feet above sea level and at −10 °C in the Himalayan mountains [14].

This garlic is also known as Kashmiri garlic or Himalayan garlic [15], and in Mexico as Japanese garlic. Recently, it was classified as *Allium ampeloprasum* L., identified through a sample confirmed by an expert from the Division of Plant Exploration and Germplasm Collection, National Herbarium and Cultivated Plants, ICAR-NBPGR, New Delhi, India, (accession number: AC-55/2021) [9]. However, *A. ampeloprasum* L. is highly diverse and widely distributed in both its wild and cultivated forms (domesticated), typically described as having large cloves, characteristic of great-headed garlic [16,17].

There have been few studies on Snow mountain garlic. Specifically, it is known that phenolics, saponins, alkaloids, triterpenes, tannins, and flavonoids are present in chloroformic and ethanolic extracts. Additionally, its antioxidant activity was observed through a 2,2-diphenyl-1-picrylhydrazyl (DPPH) radical assay, with a value of 25.64 (±0.78) µmol of Trolox Equivalent/1 g dry weight (TE/1 g dw) [18]. It has also been noted to possess fungicidal effects against *C. albicans* and *C. glabrata*, unlike *Allium sativum* L., which exhibits a fungistatic effect. Furthermore, the volatile compound cholesta-4,6-dien-3-ol, (3-beta), identified in Snow mountain garlic and not in *A. sativum* L., has been proposed as an antifungal molecule against these yeasts [19]. Moreover, in an experimental rat model, the use of Snow mountain garlic as an anti-inflammatory and antiarthritic was validated and proposed as a potential therapeutic candidate for the treatment of rheumatoid arthritis [9].

Although various species of the genus *Allium* are known to exhibit different biological effects and act as antiplatelet and antithrombotic agents [20], nothing is known about the anticoagulant effect of Snow mountain garlic.

These results lead us to consider plants used in traditional medicine as an option for managing and treating the disorders mentioned above, based on scientific evidence, which suggests they are safer. Therefore, the present study examined the *in vitro* anticoagulant effect of Snow mountain garlic applied at three different doses in plasma samples from human donors.

## 2. Results

### 2.1. Snow mountain garlic

**Figure 1:**
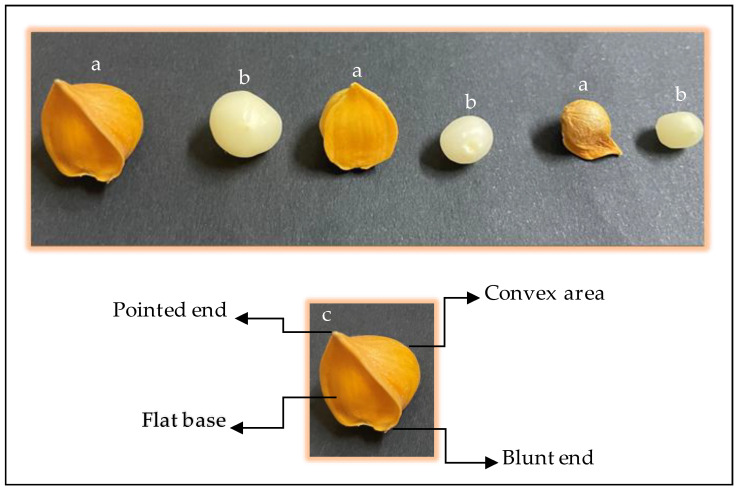
Cloves of Snow mountain garlic, (a) with shell and (b) without shell. (c) A clove of garlic with a shell; the arrows show its characteristic parts.

The cloves of Snow mountain garlic have different sizes, a hard shell cover, and a white hydrated onion-shaped structure within [18]. Figure 1 shows cloves of Snow mountain garlic purchased from the local market, which were used for the experiments in this study. Garlic cloves of different sizes, both with and without shells, can be seen in their characteristic forms (a–b). In the shelled clove, the flat base, convex area, pointed end, and blunt end are visible (c); in the shelled tooth, the circular shape and white color can be seen (b).

### 2.2. Effect of the Aqueous Extract and the Lyophilized Aqueous Extract of Snow mountain garlic on the PT and APTT in Plasma

**Figure 2:**
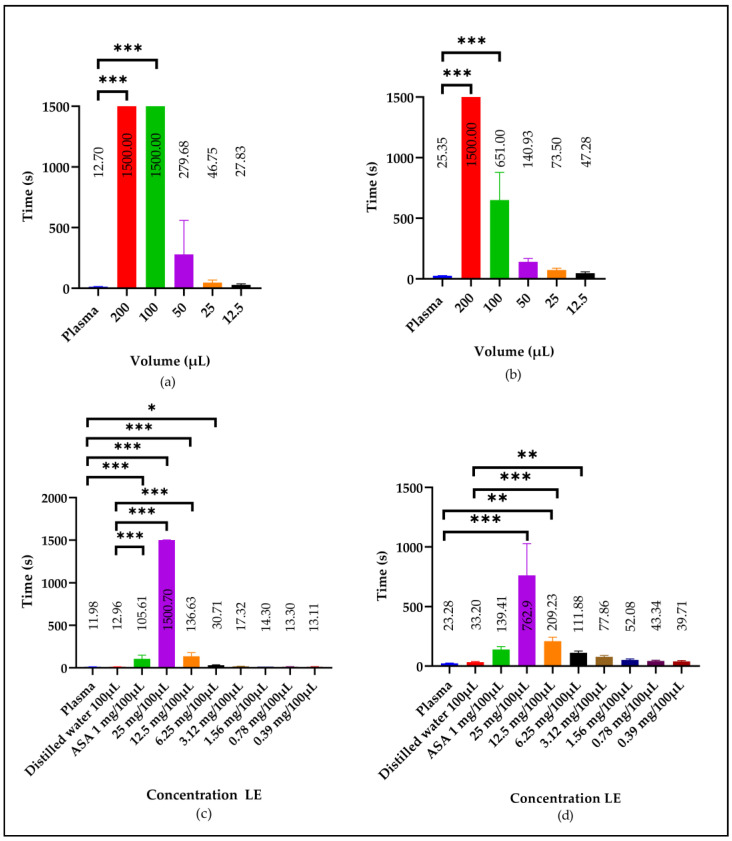
Effect of the AE of Snow mountain garlic on (**a**) the PT and (**b**) the APTT in plasma, using different volumes (μL), and the LE on (**c**) the PT and (**d**) the APTT, using different concentrations (mg/100 μL). Three donor samples were used separately in (**a**,**b**). In (**c**,**d**), 50 donor samples were used in 10 pools of five samples each. Plasma normal value: plasma; negative control: distilled water (100 µL); positive control: ASA using the concentration (1 mg/100 µL). The symbols (*), (**), and (***) indicate significant differences from the control at (*p* < 0.05, 0.01, 0.001), respectively. The determinations were made in triplicate. An ANOVA and Dunnet’s post hoc test were used to compare groups.

The aqueous extract (AE) of Snow mountain garlic affected the plasma clotting times *in vitro*. Figure 2 shows that all the volumes increased the coagulation times compared to the control. Notably, the 200 µL volume significantly increased (*p* < 0.001) the prothrombin time (PT) by 99.8 times (1500 s compared to the control: 12.7 ± 1.34 s). For the activated partial thromboplastin time (APTT), the 200 µL volume increased the clotting time by 44.2 times (1500 s compared to the control: 23.35 ± 1.29 s), which was also significant (*p* < 0.001). Notably, the experiment was stopped at 1500 s (25 min), as no clot formation was observed with 200 µL of AE; coagulation was inhibited (Figure 2a,b), and this happened in both, the intrinsic and extrinsic pathways of coagulation. No anticoagulant effect was observed with the different volumes of distilled water, which was used as a negative control. 

The effect of the AE of Snow mountain garlic was also determined using different concentrations, for which a lyophilized aqueous extract (LE) was used. Figure 1 shows a significant increase (*p* < 0.05) in the clotting time with concentrations ranging from 6.25 to 25 mg/100 μL, with the maximum effect observed at 25 mg/100 μL, resulting in a 125.2-fold increase in the PT (1500 s compared to the control: 11.98 ± 0.45 s) and an increase ranging from 21.7 to 32.75 times in the APTT (762.9 ± 256.31 s compared to the control: 23.28 ± 1.89 s; Figure 2c,d). Inhibition occurred in the intrinsic and extrinsic coagulation pathways, although the effect on the extrinsic pathway was significantly lower.

We compared the effect on the PT of the LE of Snow mountain garlic and acetylsalicylic acid (ASA), the most common anticoagulant used in the management of patients who have suffered thrombosis or are at risk of developing it. We observed that ASA had the highest anticoagulant effect and showed significant differences (105.6 ± 43.87 s) compared to the control (11.98 ± 0.45 s; *p* < 0.001; Figure 2c). Conversely, for the APTT, there was no significant difference between ASA (139.41 ± 25.34 s) and the control (23.28 ± 1.89 s; Figure 2d).

### 2.3. ED_50_ of the LE on the Plasma Clotting Times

**Figure 3:**
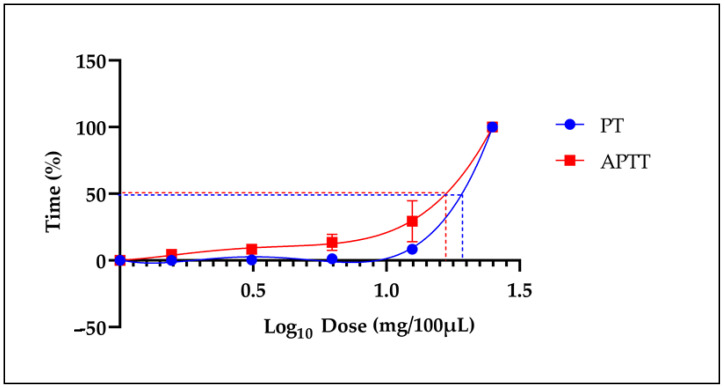
The ED_50_ of the LE of Snow mountain garlic on the plasma clotting times. The dashed lines show the ED_50_ for the PT and APTT coagulation tests.

Figure 3 shows the effective dose 50 (ED_50_) of the lyophilized extract for both the PT and APTT, which were 19.57 ± 3.76 mg/100 µL and 17.71 ± 2.38 mg/100 µL, respective.

### 2.4. Stability of the LE and AE

**Figure 4:**
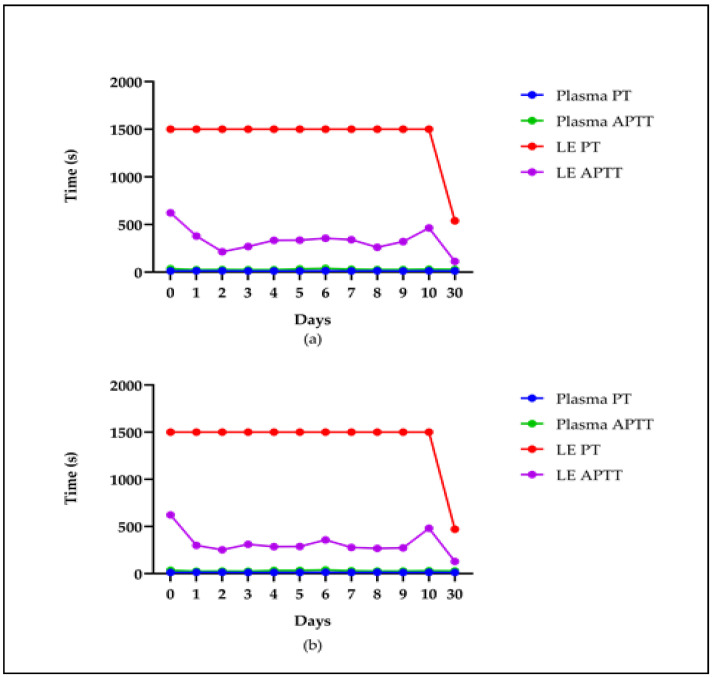
Stability of the LE at room temperature in the presence (**a**) and absence (**b**) of light. The PT and APTT times. Plasma normal value: plasma. Twenty-four plasma samples from different donors were used. The tests were conducted in triplicate.

The stability of the extract was assessed at room temperature (approximately 28 °C) over 30 days. It was observed that, regardless of whether it was shielded from light (Figure 4a) or not (Figure 4b), the extract consistently influenced the PT and APTT times in plasma for up to 10 days compared to the controls. However, by day 30, a noticeable decrease in activity was evident (Figure 4).

**Figure 5:**
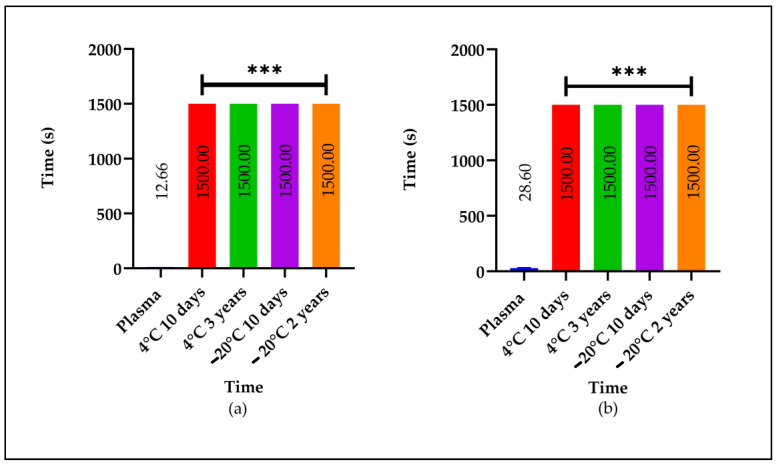
Stability of the LE at 4 °C and −20 °C and the AE at 4 °C. The (**a**) PT and (**b**) APTT times. The samples were used to determine the PT and APTT compared to a control. Three donor samples were used. Plasma normal value: plasma. The determinations were made in triplicate. The symbol (***) indicates a significant difference compared to the control at (*p* ˂ 0.001).

The stability remained consistent when the LE was resuspended in distilled water and stored at −20 °C for ten days and up to two years. Similarly, a garlic sample stored for ten days at 4 °C in the presence of light and an AE stored at 4 °C for three years exhibited a comparable stability (Figure 5).

### 2.5. Stability of the Boiled LE Sample

**Figure 6:**
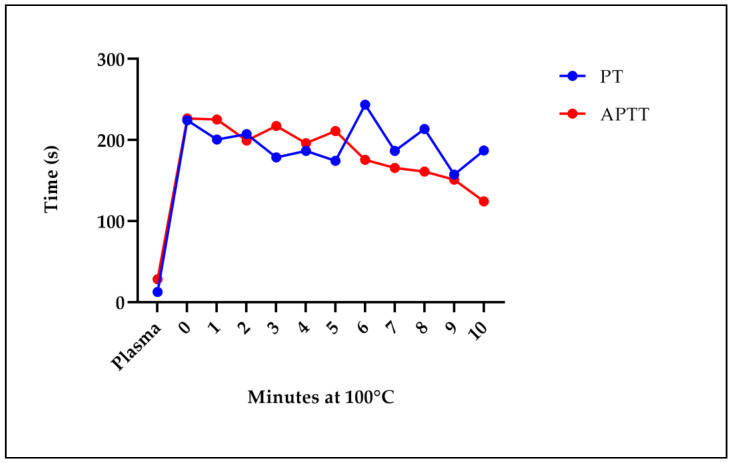
The effect on the PT and APTT in plasma after heating the extract for different times. Five plasma samples from other donors were used in a pool. Plasma normal value: plasma. The tests were conducted in triplicate, and the average value is displayed.

To determine whether the molecules responsible for the anticoagulant effect observed with the LE were proteins or metabolites, we decided to subject the extract to a heating process for up to 10 min and determine the effect on the PT and APTT in plasma. Figure 6 shows that there was a continued increase in the clotting times for the PT compared to the control. In the APTT, the curve decreased; however, a longer clotting time was still observed compared to the control. The increase in clotting times was observed from the first minute of heating, suggesting that no protein is involved in the effect, but perhaps there is a secondary metabolite.

### 2.6. Qualitative Phytochemical Profile of the LE of S. mountain garlic

Table 1 presents the qualitative findings of the phytochemical profile. The LE is rich in carbohydrates, flavones, condensed tannins, and cardiotonic heterosides, particularly coumarins or cardiotonic glycosides. Additionally, alkaloids and saponins are present.

### 2.7. Percentage of Protein and Carbohydrate of the AE and LE of Snow mountain garlic

The total protein and carbohydrate were quantified, with the LE having 3.82% and 9.45% and the AE having 4.48% and 9.68%, respectively.

### 2.8. Identification of Polyphenols by Thin-Layer Chromatography (TLC)

Six groups of metabolites are present in Snow mountain garlic (Table 1). It has been generally described in members of the genus *Allium* and other plant species that flavonoids, saponins, tannins, alkaloids, and carbohydrates have anticoagulant effects [21,22]. The isoflavones genistein and daidzein, which are obtained from soybeans, are sold commercially as products called “dietary supplements” and are used as a phytoestrogen for the management of menopause symptoms and heart disease. These isoflavones inhibited collagen-induced platelet aggregation in a dose-dependent manner [23,24] and have been found in a few quantities in *Allium sativum* L. [25].

Due to the above, we performed an extraction from the LE to determine if these compounds were present in Snow mountain garlic, which we named the isoflavone extract from the LE (IE-LE). On the other hand, through TLC, the presence of isoflavones was identified and compared with the commercial product Pronat^®^, which contains both isoflavones (daidzein and genistein). To have more solid evidence for the presence of the two isoflavones of interest in Snow mountain garlic, the samples were analyzed by HPLC-QToF-MS.

### 2.9. HPLC-QToF-MS Analysis

The results of the HPLC-QToF-MS analysis showed the presence of isoflavones, organosulfur compounds, a polyphenol, and a steroid glycoside (Table 2). The Total Ion Chromatogram (TIC) of Snow mountain garlic is in the Supplementary Material, and it indicates the possibility of the presence of different isomers or isoflavones in addition to genistein and daidzein, and other metabolites reported with anticoagulant effect; so, it was decided to determine PT and APTT using LE in human plasma.

### 2.10. Anticoagulant Effect of the IE-LE of Snow mountain garlic

**Figure 7:**
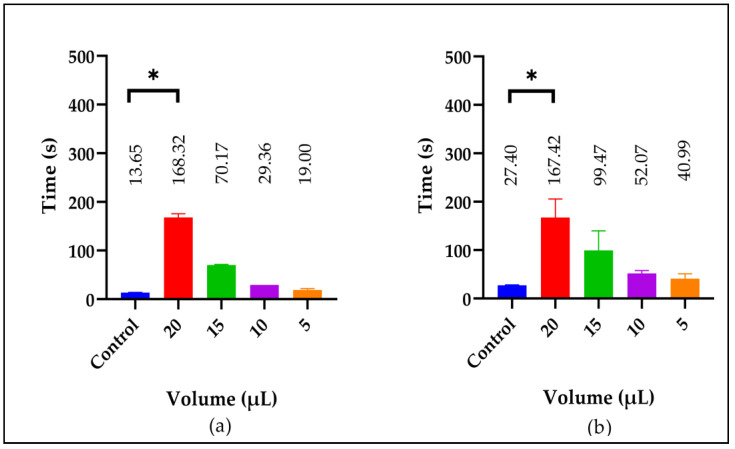
The anticoagulant effect of the Snow mountain garlic extract analyzed using HPLC-QToF-MS. The (**a**) PT and (**b**) APTT in plasma using different volumes (μL). The symbol (*) indicates significant differences compared to the control (*p* < 0.05). Donor samples were used in two pools of five samples each. Plasma normal value: plasma. The determinations were made in duplicate.

The anticoagulant effect of the IE-LE was also determined using different volumes (µL). In Figure 7, there was a significant increase (*p* < 0.05) in the clotting times at 20 µL for both the PT (168.32 ± 7.46 s) and the APTT (167.42 ± 38.15 s) compared to the control, respectively (13.66 ± 0.14 and 27.40 ± 0.77 s). The increase in clotting time was volume-dependent. Inhibition occurred in the intrinsic and extrinsic coagulation pathways. The anticoagulant effect of this extract was higher than that observed with the AE when 25 µL was used (Figure 2a,b).

### 2.11. The ED_50_ of the IE-LE of Snow mountain garlic

**Figure 8:**
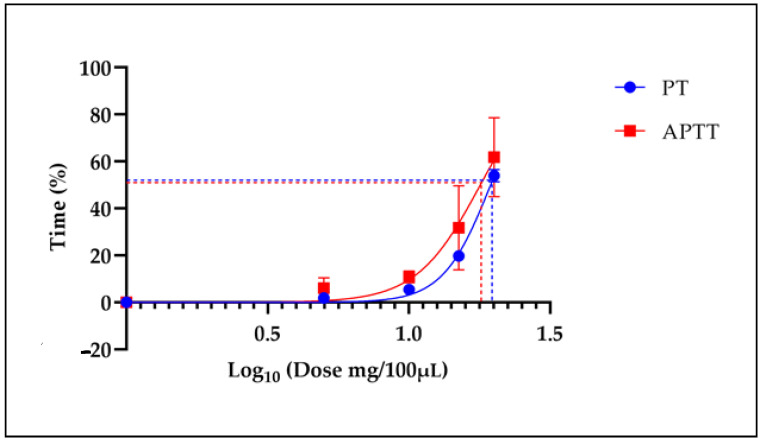
The ED_50_ of the IE-LE Snow mountain garlic on the plasma clotting times. The dashed lines show the ED_50_ for the PT and APTT coagulation tests.

Figure 8 shows the ED_50_ of the IE-LE for both the PT and APTT, which was 19.45 ± 1.86 µL and 17.71 ± 9.36 µL, respectively.

The ED_50_ is shown for the PT and APTT coagulation tests.

## 3. Discussion

In the search for new drugs to treat different diseases, new molecules with pharmacological activity have been obtained from plants. It must be remembered that at present, traditional and complementary medicines are used worldwide in many countries by the population as the first option and, on many occasions, as the only option to treat illnesses or ailments [26,27].

The traditional uses of garlic (*Allium sativum* L.) are diversified and numerous, attributed to its compounds, which are known from various studies to have diverse biological activities [20,28]. One of the effects described is the inhibition of platelet aggregation when consumed over an extended period, whether as oil, powder, raw, or aged extracts [28,29,30,31].

While *A. sativum* L. has been studied extensively, little is known about Snow mountain garlic, a phytoresource used in traditional medicine by people in the Himalayas. Specifically, in this research work, the anticoagulant effects of AE, LE, and IE-LE were scientifically studied *in vitro*, compared to a negative control (without garlic extract). We tested the lengthening of clotting times with the three extracts through the intrinsic and extrinsic coagulation pathways. In general, automatic coagulometers detect the first strand of fibrin that forms through the laser beam emitted by the device, which triggers an alarm for the evaluator to remove the tray and verify the clot formation. The time between placing the cuvette in the device and the alarm sounding is recorded, indicating whether the clotting time is normal. Typically, clot formation is rapid, and, by the time the alarm sounds, a consistent clot is formed, observable by the evaluator. However, this was not the case in the presence of the AE and LE; the clot did not have a firm consistency. Instead, it was rather weak, suggesting that the effect occurs by delaying the union of fibrin molecules or interfering with the fibrin polymerization into fibers, thus delaying clot formation, which could impact platelet aggregation in a complete system [32].

Garlic essential oil, alcoholic extracts, aged garlic, garlic paste, and ethyl acetate, as well as garlic powder and garlic oil rich in diallyl trisulfide, aqueous extract, and others have shown to have anticoagulant effects in various studies, with some tested *in vitro* and others *in vivo* [33,34,35,36,37,38,39,40,41]. Similarly, the effect of the aqueous extract of *A. sativum* on *in vitro* coagulation has been reported, indicating that the thrombin time was 7.32 min with a dose of 500 µg/mL [36]. None of these studies provide an effective way to use garlic, partly due to the limited number of samples, the type of extract, and the lack of knowledge about the molecule or molecules responsible for the effect.

In our study, the anticoagulant effect of Snow mountain garlic was determined using the PT and APTT tests, and it was observed from 6.25 mg/100 µL for the PT, with a maximum effect at 25 mg/100 µL, which was much higher than what was reported for *A. sativum* L. However, the time was 1500 s = 25 min, although the clot never really formed. It is important to consider that the tests used in both studies differed. For the APTT, the same effect was observed with 25 mg/100 µL of LE. The ED_50_ obtained sets the standard for studies from which a human herbal dose could be suggested. There are few studies related to the anticoagulant effect using aqueous extracts. A relevant piece of information acquired from our research was the stability of the extracts, as this could allow for usage without needing a fresh extract daily and without refrigeration or freezing. Additionally, it could be used in gel capsules stored at room temperature. We were unable to locate other studies related to the stability of garlic extracts.

Regarding the chemical character of the molecule(s) with anticoagulant activity, the fact that the activity was preserved even after heating the sample for 10 min indicated that it was not a protein. The HPLC-QToF-MS analysis of the IE-LE showed the presence of metabolites with anticoagulant activity. The phytochemical profile of the LE showed the presence of antioxidants such as flavones. Flavonoids, as secondary metabolites of countless plants, act as powerful antioxidants and natural anti-inflammatories. They have also been described as acting as anticoagulants [42,43].

The pathological process opposite to hemostasis is thrombosis. Primary hemostasis begins with platelet adhesion and activation, which triggers secondary hemostasis through the activation of coagulation factors. Therefore, it has been proposed that plant extracts rich in flavonoids could be used to prevent and treat coagulation problems, such as the formation of thrombi and emboli in blood vessels [44]. The results obtained through computational docking showed that alliin inhibits nitric oxide synthase, an enzyme that in its reaction produces nitric oxide, which inhibits platelet aggregation [45]. Inducible nitric oxide synthase modulated the endothelin-1-dependent release of prostacyclin and the inhibition of platelet aggregation ex vivo in mice [46].

In a recent study, it was observed that ajoene is a potent platelet inhibitor that prevents the binding of fibrinogen to the thromboxane TPR and thrombin PAR 1 receptors [47]. Additionally, it has been established that the compounds ethyl ethane thiosulfinate and ethyl methane thiofulfate, found in Snow mountain garlic in the present work, inhibit platelet aggregation by 89–90% and are more potent than aspirin in similar concentrations [48].

It has been reported that isoflavones such as daidzein, used in collagen-induced platelets, suppress the production of thromboxane A2 by inhibiting cytosolic phospholipase A2 (cPLA2), without affecting COX1. These molecules also attenuate the PI3K/PDK1/Akt/GSK3 αβ and MAPK (p38, ERK) signaling pathways and increase the phosphorylation of inositol triphosphate receptor 1 (IP3R1) and vasodilator-stimulated phosphoprotein (VASP), as well as the level of cyclic adenosine monophosphate (cAMP). This implies that they could inhibit the release of ATP, serotonin, and P-selectin granules and the activation of αIIbβ3 integrin, consequently inhibiting clot retraction [49] and directly impacting the inhibition of coagulation. Furthermore, using an ex vivo perfusion chamber model, it was demonstrated that genistein and daidzein have an inhibitory effect on thrombus formation under a high shear force of 1800 s^−1^. These isoflavones interact with 14-3-3ζ, an adapter protein that regulates platelet activation mediated by GpIb-IX and αIIbβ3; platelet propagation mediated by integrins; and signal transduction via external stimuli [21]. Glycitein, another isoflavone, has also been shown to inhibit platelet aggregation [50].

Similarly, another study developed in 2021 explained that oxidative stress increases the adhesion of platelets to blood vessels, promoting vascular pathology in neurodegenerative diseases. This study indicated that rosmarinic acid, a polyphenol found in the present work in Snow mountain garlic, inhibits Aβ 1–40-induced platelet adhesion through NADPH oxidase/ROS/PKC-δ/integrin α IIb β signaling [51].

Furthermore, a phytosterol, β-sitosterol-3-O-β-D-glucopyranoside, found in the wild garlic *Allium ursinum* and identified in this study in Snow mountain garlic, inhibits ADP-induced platelet aggregation in human blood platelets [52].

Finally, it was found that the metabolites of Snow mountain garlic have an anticoagulant effect. In the scientific literature, the anticoagulant effect is carried out at the level of platelet aggregation; however, there is a lack of scientific evidence that explains whether an interaction occurs with the coagulation factors of secondary hemostasis.

The anticoagulant effect of the IE-LE of Snow mountain garlic was determined, which was higher with 20 µL of this extract than that observed with 25 µL of the AE. This can be attributed to the fact that molecules with anticoagulant capacity were concentrated in the IE-LE extract. Most likely, they all act simultaneously and at different levels, giving rise to a stronger effect.

Most of the studies have focused on the properties of sulfur compounds specific to the genus *Allium* [47]. Recently, a study on *Allium ampeloprasum* var. *Holmense* observed that it had more polyphenols and less organosulfur than *Allium sativum* L. [53].

Therefore, the identification of the isoflavones genistein and daidzein in Snow mountain garlic could be a novel area of research, with the potential to open new paths in understanding their pharmacological properties.

Moreover, the joint anticoagulant activity of the metabolites identified in IE-LE (alliin, ajoene, genistein, glycitein, rosmarinic acid, ethyl methane thiosulfate, ethyl ethane-thiosulfinate, daidzein, and β-sitosterol-3-O-β-D-glucopyranoside) has not been previously described in the literature of the Snow mountain garlic.

Snow mountain garlic is a species of *Allium* used in the trans-Himalayan traditional medicine system [9]. The anticoagulant effect observed with this garlic opens the door for its possible use in the preventive control of thromboembolic diseases and for the management of patients who present health problems of this type.

It will be important in the future to consider personalizing the dosage of the compound(s), depending on the cardiovascular alteration and the site of action of the molecules involved as anticoagulants, which may be acting individually or synergistically. Genetic variation in the efficacy and safety of medications has become one of the pillars of pharmacological development; therefore, it will be important to take into account the contribution of pharmacogenomics in short-term studies on the anticoagulant effect of Snow mountain garlic. It is known that genetic variations in the pharmacological receptors or in the genes involved in the distribution of the drug generate different clinical results and responses in patients treated with the same drug, as the joint action of numerous genes can result in a unique response to a drug, as determined in the field of pharmacogenetics [54].

## 4. Materials and Method

### 4.1. Plant Material

The Snow mountain garlic was obtained from a local market on Reforma Street 405A, Historical Center, 78000 San Luis Potosi, Mexico. Taxonomic identification was performed by biologist Eleazar Carranza at the Herbarium “Isidro Palacios”, Desert Area Research Institute, Autonomous University of San Luis Potosi, using the World Flora Online database to identify the scientific name of Snow mountain garlic, *Allium ampeloprasum* L. (accessed on 17 March 2024). Furthermore, a first batch of the plant was prepared and analyzed by the same expert, who assigned the classification voucher (Table 3). We acquired a 5 kg batch to carry out the present work.

### 4.2. Chemicals

The chemicals used were as follows: sulfuric acid (J.T. Baker^®^, CDMX, Mexico), chlorotic acid (CTR scientific^®^, MTY, Mexico), acetonitrile (Tedia the pure choice, Fairfield, OH, USA), acetonitrile for HPLC-QToF-MS analysis (Honeywell Burdick and Jackson, Morristown, NJ, USA), methanol and ammonium formate (CTR scientific, MTY, Mexico). 

### 4.3. Obtaining the Aqueous Extract

To obtain the AE, Snow mountain garlic bulbs that are not dry, without damage to the peel, with a homogeneous color and characteristic odor were selected. Afterward, a 50 g sample of Snow mountain garlic without peel was weighed on a garnet scale (Ohaus, Triple Beam 700/800 series, Parsippany, NJ, USA). The shelled cloves were disinfected with absolute ethanol. Once the alcohol had evaporated, they were crushed in a clean porcelain mortar, slowly adding 40 mL of distilled H_2_O until a homogeneous paste was formed; then, this was filtered with No. 0 filter paper (no brand, SLP, Mexico) using a plastic funnel. The filtrate was stored in tubes (Eppendorf, Hamburg, Germany) in 1 mL aliquots and frozen at −20 °C until use. This was named the AE.

### 4.4. Obtaining the Lyophilized Aqueous Extract

The procedure described previously was followed to obtain the aqueous extract as described in Section 4.3. Each 20 mL of the aqueous extract was placed in a 50 mL Falcon tube (Capp, Nordhausen, Germany), frozen at −30 °C, and then lyophilized for 72 h. (FreeZone Labconco of 2.5 L, Kansas City, MO, USA). This was stored at −30 °C until use and named the LE.

### 4.5. Determination of the Coagulation Times

#### 4.5.1. Volunteers 

**Figure 9:**
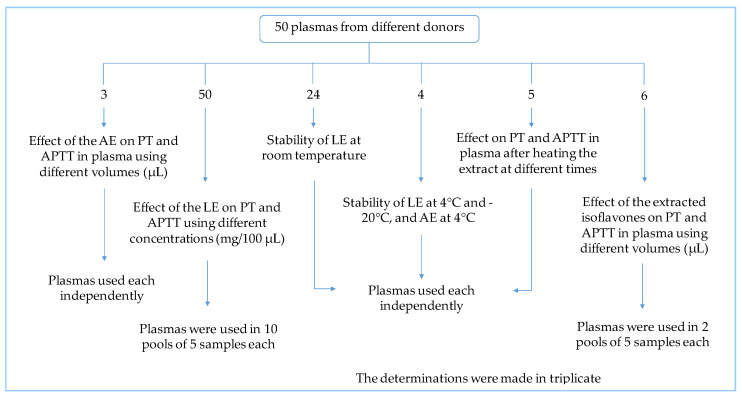
Scheme showing the use of plasma samples donated by the participants in the study.

A total of 50 volunteers participated in the study; they were over 18 years of age and had not used any known anticoagulant or antiplatelet drugs. A signed informed consent letter was obtained from all participants before collecting the blood samples and after an explanation of the objectives of the research. This study was conducted based on the specifications of the General Health Law Regulations on Research in Mexico, and the principles of the Declaration of Helsinki were followed throughout the study [55,56]. The Research Ethics Committee of the Faculty of Nursing and Nutrition at the UASLP (CEIFE-2018-258) approved the project. Plasma samples were used as shown in Figure 9.

#### 4.5.2. Obtaining Blood Samples from Volunteers

First, 5.4 mL of peripheral venous blood was obtained, according to the standard technique, in 2.7 mL BD Vacutainer^®^ tubes (Franklin Lake, NJ, USA) with 0.5 3.2% sodium citrate (2 per donor). The samples were centrifuged for 10 min at 1942× *g* to obtain the plasmas; later, they were separated into Eppendorf tubes. The plasmas were frozen at −30 °C until use. The blood samples were obtained in the clinical analysis laboratory of the Faculty of Nursing and Nutrition of the Autonomous University of San Luis Potosi. Biological waste management was carried out as established by the Official Mexican Standard NOM-087-ECOL-SSA1-2002, Environmental protection—Environmental Health—Biological–Infectious Hazardous Waste—Classification and Management Specifications [57].

#### 4.5.3. Testing the PT and APTT with Control Plasma

The clotting times were measured as the PT and APTT. For this purpose, BFT II (Dade Behring, Marburg, Germany) equipment (automated method) was used. The analyzer automatically recognizes the cuvette placement and provides the results for each test. The principle is based on measuring the optic changes when the plasma inside the equipment forms clots; the tests results occur in seconds.

Determination of the PT was made using the SPINREACT kit (Girona, Spain); briefly, 50 μL of plasma was incubated at 37 °C for 1 min, and 100 μL of the prothrombin time reagent was added. The coagulometer timer was automatically started to measure the clot formation time from the moment at which the reagent was added. The value considered normal was from 10 to 15 s. Each test was conducted in triplicate.

Determination of the APTT was made using the SPINREACT kit (Girona, Spain). At room temperature, 100 μL of the activator (ellagic acid or R1) was added to 100 μL of human plasma. They were mixed and incubated at 37 °C for 5 min (activation time), with 100 μL of the initiator (calcium chloride or R2) added later. The coagulometer timer was automatically started to measure the clot formation time from the moment the R2 was added. The value considered as normal was from 20 to 40 s. Each test was conducted in triplicate.

#### 4.5.4. Testing the Effect of the AE of Snow mountain garlic on the PT and APTT in Plasma

The PT and APTT tests were performed by incubating human plasma with the AE at different volumes (200, 100, 50, 25, 12.5 μL). The procedure is described in Section 4.5.3. The controls were the following: normal, the PT and APTT value of each volunteer; negative, distilled water (200, 100, 50, 25, 12.5 μL). Each test was performed in triplicate.

#### 4.5.5. Testing the Effect of the LE of Snow mountain garlic on the PT and APTT in Plasma

To test the effect on the clotting times, 1 g of LE was resuspended in 4 mL of distilled water, obtaining a concentration of 250 mg/mL. From this, dilutions were made for the following concentrations: 25, 12.5, 6.25, 3.125, 1.562, 0.781, and 0.39 mg/100 μL. The PT and APTT tests were conducted as described in Section 4.5.3. Ten pools of plasma, from 5 different volunteers each, were made (50 volunteers). Each pool was obtained by mixing 1 mL of 5 plasmas. The controls were the following: normal, the PT and APTT value of each plasma pool; negative, distilled water 100 μL; positive, the ASA at a concentration of 1 mg/100 µL, which was the minimum concentration that could be dissolved due to the presentation of the drugs available. Each test was performed in triplicate.

### 4.6. The LE and AE Stability

The extract stability was measured by testing the effect on the PT and APTT in plasma. For this purpose, 10 g of LE was resuspended in 40 mL of distilled water. Afterward, the volume was divided into 4 aliquots of 10 mL each. Two were kept at room temperature, with one exposed to light and the other protected from light for 10 days. The third aliquot was stored at 4 °C and the fourth at −20 °C. The stability was measured for 10 consecutive days. Each test was performed in triplicate using 11 plasma samples from different donors. Then, an LE resuspended in distilled water that had been stored for 2 years at −20 °C and another AE that had been stored at 4 °C for 3 years were tested to determine whether they possessed anticoagulant activity.

### 4.7. Determination of the Stability of the Boiled LE Sample

To determine whether the molecule(s) responsible for the anticoagulant effect observed with the LE of Snow mountain garlic were proteins or metabolites, 10 aliquots of 1000 µL were placed in boiling water and removed one by one every minute for a total of 10 min. Each sample was placed on ice immediately after being removed from the boiling water. After that, the tubes were centrifuged for 10 min at 1942× *g* (HERMLE Labortechnik GmbH model Z216MK microcentrifuge, Wehingen, Germany). Then, 100 µL of the supernatant was used to determine the effect on the PT and APTT in plasma, as described in Section 4.5.3. Each test was performed in duplicate.

### 4.8. Qualitative Phytochemical Profile of the LE of S. mountain garlic

The qualitative phytochemical profile was measured using 0.5 g of LE mixed in 20 mL of distilled water. Determinations were made for the identification of groups of secondary metabolites through a preliminary phytochemical study, which involved precipitation and coloration reactions as described by Bruneton [58], Domínguez [59], and Trease and Evans [60].

To recognize the presence of flavones, the reactions of flavonic heterosides were studied: Constantinesscu, Shinoda, Dimroth (5-hydroxy flavones), sulfuric acid, leucoanthocyanins (flavandiol 3,4), and anthocyanins. For saponins, Liebermann–Burchard, Rosenthaler, and foam production tests were used. For sesquiterpene lactones, the ferric hydroxymate reaction was performed. The differentiation test was carried out for tannins between hydrolyzable and condensed tannins with ferric salts, precipitation with Stiasny reagents, the oxidation of catechin tannins, a catechin test, a chlorogenic acid test, and a gallic acid test. A general reaction was conducted to determine the coumarins, while the Baljet, Legal, Raymond, and Keller–Killani tests were carried out for the cardiotonic heterosides. The alkaloids were determined by the Meyer lump, Wagner, Hager, Dragendorff, and Sonnenchein reactions. The direct extraction of free genin forms and the extraction before oxidative acid hydrolysis were conducted to assess anthraquinone heterosides. The quinones were identified through a general reaction and the free anthraquinone derivatives through the Börntrager test. Carbohydrates were determined using the Fehling and Molisch reactions [61,62]. The qualitative criteria for the phytochemical analysis were based on Clavijo Moreno and Cruz Jaramillo (2017) [63].

### 4.9. Protein and Carbohydrate Quantification of the AE and LE of S. mountain garlic

To determine the protein concentration, the Kjeldahl microscale method was used with a SEV-PRENDO brand micro digester (8U, PUE, Mexico) and a SEV-PRENDO micro distiller (DEK-01, PUE, Mexico) [64] and, to determine the total sugars, the Lane–Eynon volumetric method was used [65].

### 4.10. Isoflavone Extraction from S. mountain garlic LE

Isoflavones were extracted as described by Wang and Murphy (1994). Briefly, 500 mg of LE from Snow mountain garlic was added to 2 mL of 0.1 N HCl and 10 mL of acetonitrile and mixed for 2 h at room temperature on a plate at 300 rpm using a magnetic stirrer. Subsequently, the mixture was filtered through Whatman™ No. 42 filter paper (GE healthcare life sciences, Shanghai, China) and evaporated under vacuum at 25 °C, using a rotary evaporator (BUCHI R-3, St. Gallen, Switzerland) [66]. The presence of isoflavones was verified via TLC (silica gel Merck 60F254 de 10 × 5 cm, Darmstadt, Germany), using the technique described by Sabha (2011). First, 3 µL of the isoflavones extracted from Snow mountain garlic was placed 1 cm from the base of the plate. The eluent used was ethyl acetate/methanol/water (77:13:10) [67]. Isoflavones from a commercial product (Pronat^®^, CDMX, Mexico) were used as a reference.

Once the TLC development was completed in a glass chamber pre-saturated with the eluent, the plate was removed, air-dried at room temperature, and sequentially immersed in two developer solutions. The first solution consisted of 1 g of vanillin dissolved in 100 mL of methanol, followed by immersion in a second solution of 10 mL of sulfuric acid mixed with 100 mL of methanol. After drying, the plate was heated at 110 °C for 10 min. Flavonols and flavons appeared as deep yellow spots and blurred in UV (254 nm), while proanthocyanidins appeared as intense red spots [67]. We named this isoflavone extract from Snow mountain garlic LE (IE-LE).

### 4.11. Testing the Effect of the IE-LE of S. mountain garlic on the PT and APTT in Plasma

To determine the effect on the clotting times, we composed the dose–response curve with the following volumes: 20, 15, 10, and 5 µL. The PT and APTT tests were conducted as described in Section 4.5.3. For these, pools of plasma were created by combining samples from 5 different volunteers, resulting in 2 distinct pools (10 participants in total). Each pool was prepared by mixing 1 mL of plasma from each of the 5 individuals. Each test was performed in duplicate.

### 4.12. HPLC-QToF-MS Analysis of the IE-LE of S. mountain garlic

Analysis of IE-LE by HPLC-QToF-MS was performed using a Kinetex Phenyl-hexyl 100 A 150 × 46 Core Shell column, (Phenomenex, Torrance, CA, USA). The samples were injected directly (extraction of LE from Snow mountain garlic and isoflavones from commercial products). The mobile phase consisted of (A) H_2_O + 0.5 mM ammonium formate and (B) ACN+ 0.5 mM ammonium formate. The flow rate was 1 mL/min, and the gradient conditions were as follows: 0–20 min, 10–90% B; 20.1–25 min 90% B; 25.1–30 10% B. The column temperature was kept at 25 °C. The ESI ionization mode was carried out in a 1260-Infinity ll liquid chromatograph with a diode array detector (Agilent, Santa Clara, CA, USA) and a QToF-MS mass detector (Agilent, Santa Clara, CA, USA) using the following conditions: breaker voltage 175 V, negative polarity, m/Z range of 50–1000. The calibration was performed with an ESI-L low concentration, Tunning Mix G1969-85000; batch 0006776102El (Agilent, Santa Clara, CA, USA). The injection volume was 100 μL of the IE-LE of Snow mountain garlic. The results are shown in the Total Ion Chromatogram (TIC) (Supplementary Material). MassHunter Workstation software version B.08.00 Data Acquisition software (Agilent Technologies, Santa Clara, CA, USA) was utilized for data collection. The ions of interest were specifically searched by their formula, obtaining the Extracted Ion Chromatograms (EIC).

### 4.13. Statistical Analysis and Calculation of the ED_50_

The *in vitro* LE anticoagulant effect was analyzed using SPSS 26 and GraphPad Prism 8 software. A one-way ANOVA was performed to identify global differences between groups, followed by post hoc analyses using Tukey, Games–Howell, and Dunnett tests for pairwise comparisons. The anticoagulant effect of the Snow mountain garlic extract, analyzed using HPLC-QToF-MS, was determined using the Friedman test and Dunn’s post hoc test.

The ED_50_ of the LE and IE-LE was calculated using GraphPad Prism 8 software by performing a non-linear regression with normalized clotting times and logarithmic transformations of doses (base 10). The regression equation used was a fourth-degree polynomial for both the PT and APTT tests.

## 5. Conclusions

The AE and LE of Snow mountain garlic showed anticoagulant activity *in vitro*. Additionally, we demonstrated stability at different times and temperatures.

Isoflavones, organosulfur compounds, polyphenols, and steroid glycosides show anticoagulant activity *in vitro* or *in vivo*. We identified, by HPLC-QToF-MS, compounds like these (alliin, ajoene, genistein, glycitein, rosmarinic acid, ethyl methane thiosulfate, ethyl ethane-thiosulfinate, daidzein, and β-sitosterol-3-O-β-D-glucopyranoside) in the Snow mountain garlic extract.

The IE-LE of Snow mountain garlic has anticoagulant activity *in vitro.*

Future studies on the mechanism of action (both in silico and *in vitro*) of the identified molecules would significantly advance the scientific understanding of the medicinal properties of Snow mountain garlic.

It would also be important to consider the contributions of pharmacogenomics and pharmacogenetics for future research, to propose personalized dosing, which is the current trend in pharmacology.

The present study is the first to establish the anticoagulant effect of Snow mountain garlic. However, before proposing its use as a treatment or as part of a treatment to prevent thromboembolic diseases, it is essential to conduct preclinical studies in rats using the LE of Snow mountain garlic, to determine whether the anticoagulant effect is preserved in a complete system, which could bring us closer to the mechanism of action and support the development of a scientifically based herbal dosage.

## Figures and Tables

**Table 1:** *S. mountain garlic* LE phytochemical profile.

Metabolite	Reaction	Result
Carbohydrates	Molisch reaction	+
Flavones	Flavone heterosides	+
Saponins	Foam test	+++
Tannins	Condensed tannins: differentiation between hydrolyzable and condensed tannins with iron salts.	+
	Catechin tannins: oxidation of catechin tannins	+++
Cardiotonic heterosides	Baljet reaction, Legal, Raymond	+
Alkaloids	Wagner, Dragendorff, and Sonnenchein precipitate	++

Slightly positive (+); positive (++); very positive (+++).

**Table 2:** Compounds identified using HPLC-QToF-MS in the IE-LE of Snow mountain garlic.

tR (min)	Compound (Metabolite)	Formula	[M-H]^−^
1.76	Alliin (organosulfur compound)	C_6_H_11_NO_3_S	176.0387
2.17	Ajoene (organosulfur compound)	C_9_H_14_OS_3_	233.0134
11.70	**Genistein (isoflavone)**	C_15_H_10_O_4_	253.0506
12.65	Glycitein (isoflavone)	C_16_H_12_O_5_	283.0612
12.88	Rosmarinic acid (polyphenol)	C_18_H_16_O_8_	359.0772
15.60	Ethyl methane thiosulfate (organosulfur compound)	C_3_H_8_O_3_S	123.0121
15.64	Ethyl ethane- thiosulfinates (organosulfur compound)	C_4_H_10_O_3_S	137.0278
16.54	**Daidzein (isoflavone)**	C_15_H_10_O_4_	253.0506
26.22	β-si-tosterol-3-O-β-D-glucopyranoside (steroid glycoside)	C_35_H_60_O_6_	575.4317

tR = time retention; [M-H]^−^ = adduct for negative ion.

**Table 3:** Taxonomic classification of *Allium ampeloprasum* L.

Kingdom	Plantae
Phylum	Magnoliophyta
Class	Liliopsida
Order	Asparagales
Family	Amaryllidaceae
Genus	Allium
Species	Ampeloprasum
Common name	Snow mountain garlic, Himalayan garlic, Kashmiri garlic
Voucher	70,262

## Data Availability

Data will be available by request; a copy of the databases for statistical analysis to obtain ED_50_ and data on clotting times are available from the senior author I.C.-M. and Y.T.-F.

## Supplementary Materials

The following supporting information can be downloaded at: https://www.mdpi.com/article/10.3390/molecules29204958/s1.
