# Supplementary figures and images for: Anticoagulant Effect of Snow mountain garlic: In Vitro Evaluation of Aqueous Extract
Source: Molecules. 2024 Oct 20;29(20):4958. doi: 10.3390/molecules29204958 (PMC11510279; doi:10.3390/molecules29204958)

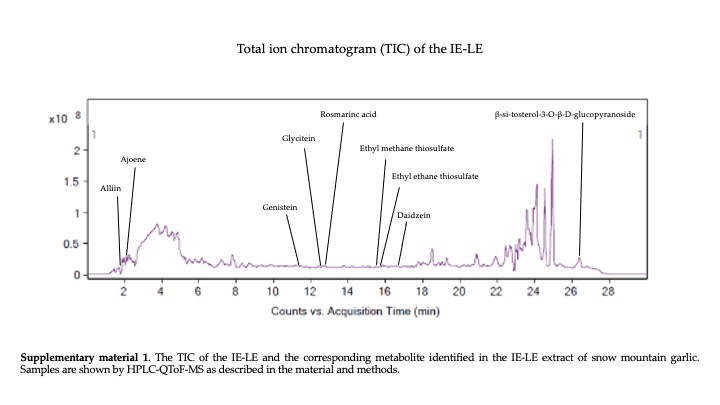

Supplement: Supplementary file 1 [file molecules-29-04958-s001.zip › molecules-3222009-supplementary.jpeg]
